# Vagal Nerve Stimulation Attenuates Ischemia-Reperfusion Induced Retina Dysfunction in Acute Ocular Hypertension

**DOI:** 10.3389/fnins.2019.00087

**Published:** 2019-02-11

**Authors:** Meng-Nan Jiang, Yu-Yang Zhou, Di-Hao Hua, Jia-Yi Yang, Man-Li Hu, Yi-Qiao Xing

**Affiliations:** ^1^Eye Center, Renmin Hospital of Wuhan University, Wuhan, China; ^2^College of Veterinary Medicine, Huazhong Agricultural University, Wuhan, China

**Keywords:** vagal nerve stimulation, retina, ischemia/reperfusion, neuroprotection, inflammation, vasoactive intestinal polypeptide

## Abstract

**Purpose:** The present study aimed to investigate whether cervical vagal nerve stimulation (VNS) could prevent retinal ganglion cell (RGC) loss and retinal dysfunction after ischemia/reperfusion (I/R) injury.

**Methods:** First, rats were randomly divided into sham group (*n* = 4) and VNS group (*n* = 12). Activation of the nodose ganglia (NOG), nucleus of the solitary tract (NTS), superior salivatory nucleus (SSN), and pterygopalatine ganglion (PPG) neural circuit were evaluated by c-fos expression at 0 h after sham VNS and at 0 h (*n* = 4), 6 h (*n* = 4), 72 h (*n* = 4) after VNS. Secondly, rats were randomly assigned to I/R group (pressure-induced retinal ischemia for 1 h and reperfusion for 1 h in the right eye, *n* = 16) and I/R+VNS group (right cervical VNS for 2 h during the I/R period, *n* = 16). The left eye of each rat served as a control. Electroretinogram (ERG), RGC numbers, tumor necrosis factor-α (TNF-α) and vasoactive intestinal polypeptide (VIP) levels in retina were determined. Additionally, the level of VIP in PPG was evaluated.

**Results:** In the first part of the study, compared with the sham group, the VNS group exhibited significantly increased expression of c-fos in NOG, NTS, SSN, and PPG tissues at 0, 6, and 72 h. In the second part of the study, compared with left eyes, retinal function in right eyes (as assessed by the a-wave, b-wave and the oscillatory potential amplitudes of ERG and RGC data) was significantly decreased by I/R. The decreased retinal function was attenuated by VNS. In addition, I/R induced an increase in inflammation, which was reflected by elevated TNF-α expression in the retina. VNS significantly attenuated the increase in I/R-induced inflammation. Moreover, VIP expression in the retina and PPG, which may contribute to the inhibition of the inflammatory response, was significantly increased after VNS.

**Conclusion:** VNS could protect against retinal I/R injury by downregulating TNF-α. Upregulation of VIP expression due to activation of the NOG-NTS-SSN-PPG neural circuit may underlie to the protective effects of VNS.

## Introduction

Retinal ischemia/reperfusion (I/R) injury is a common pathological process that develops in a variety of retinal disorders including diabetic retinopathy, retinopathy of prematurity and glaucoma, resulting in the progressive loss of retinal ganglion cells (RGCs) and vision (Kaur et al., 2008; Narayanan et al., 2013; Palmhof et al., 2018). Acute elevation of intraocular pressure (IOP) followed by reperfusion was a well-established model used to investigate the mechanisms of, and potential therapy for retinal ischemia (Zheng et al., 2007; Mi et al., 2012; Palmhof et al., 2018). Chi et al. (2014) used an acute IOP model to investigate the underlying mechanism of RGC death and found that IOP increases IL-1β expression and RGC death. Kim et al. (2017) and Munemasa and Kitaoka (2012) found that expression of pro-inflammatory cytokines, such as tumor necrosis factor-α (TNF-α), is significantly increased after retinal I/R injury and these increased cytokines can cause axon degeneration and RGC loss. These researches have indicated that an increased inflammatory response promotes neuron damage during retinal I/R injury. Although previous studies have shown that some medicines or operation can remit retinal reperfusion (I/R) injury (Adams et al., 2018; Le et al., 2018), the side effects and shortcomings of present therapy highlights the need for improved therapies.

It has been reported that vagal nerve stimulation (VNS) can protect against I/R injuries in multiple organs, including the heart, kidney and cerebra, through its anti-inflammatory properties (Zhao et al., 2013; Inoue et al., 2016; Xu et al., 2018). The central vagal nerve center and nucleus of solitary tract (NTS) can project parasympathetic nerves to the retina and choroid through the superior salivatory nucleus (SSN) and pterygopalatine ganglion (PPG) neural circuit (Agassandian et al., 2003; Chunyan et al., 2015; McDougal and Gamlin, 2015; Li et al., 2016b). Several studies have shown that the parasympathetic nerve in these neural circuits can synthesize and release the anti-inflammatory peptide-vasoactive intestinal peptide (VIP) to protect the retina (Chandrasekharan et al., 2013; Chen L. et al., 2015; Ganea et al., 2015; Cakmak et al., 2017; Atlasz et al., 2018). These studies suggested that activation of the NTS-SSN-PPG circuit could promote the release of vasoactive intestinal peptide (VIP) However, whether VNS can protect the retina against I/R injury through its anti-inflammatory property remains unknown.

In the present study, the IOP-induced retinal ischemia model was applied to observe the effect of VNS on electroretinogram (ERG), retinal ganglion cells (RGCs) and the expression level of VIP and TNF-α in the PPG and retina. We hypothesize that VNS could protect the retina against I/R injury by regulating VIP and TNF-α via activation of the NTS-SSN-PPG pathway.

## Materials and Methods

### Animal Preparation

Male SD rats (220 ± 20 g, supplied by the Experimental Animal Center of Renmin Hospital, Wuhan University, China) were fed a standard diet, provided water randomly and kept on a 12-h light/12-h dark cycle. All experimental procedures were performed in accordance with the ARVO Statement for the Use of Animals in Ophthalmic and Vision Research. The experimental protocol was approved by the Ethics Committee of Renmin Hospital, Wuhan University.

### Establishment of the Retinal Ischemia/Reperfusion (I/R) Model

Rats were anesthetized with an intraperitoneal (i.p.) injection of xylazine (10 mg/kg) and ketamine (100 mg/kg). 1% tropicamide was then used to dilate the pupils, and 0.5% tetracaine hydrochloride was used to topically anesthetize the corneas. A 30-gauge needle was cannulated into the right anterior chamber and connected to a 0.9% saline reservoir, which maintained an IOP of 90 mmHg for 1 h, followed by reperfusion for 1 h (Pinar-Sueiro et al., 2013; Liu et al., 2016; Wang W. et al., 2018). The success of the model was confirmed by a tonometer and by observing the loss of the red reflex. Visualization of the red reflex following needle withdrawal served as an indication of retina reperfusion (Fang et al., 2013; Chi et al., 2014; Hashem et al., 2017). The other eye remained untreated and served as a control. Rats were placed on a temperature-controlled heating pad to maintain body temperature at 37°C throughout the whole surgical intervention.

### Vagal Nerve Stimulation

A ventral midline incision was made on the neck, then, the muscles were retracted, and the right cervical vagal nerve was isolated from the right carotid sheath. VNS (5 Hz, 1 ms pulse width, 2 s interval) was applied via a pair of Teflon-coated silver hooks connected to a stimulator (S20, Jinjiang, Chengdu City, China). Surface ECG was continuously recorded during VNS. The stimulation level was defined as the electric current required to cause a 10% decrease in heart rate.

### Experiment Protocol

**FIGURE 1:**
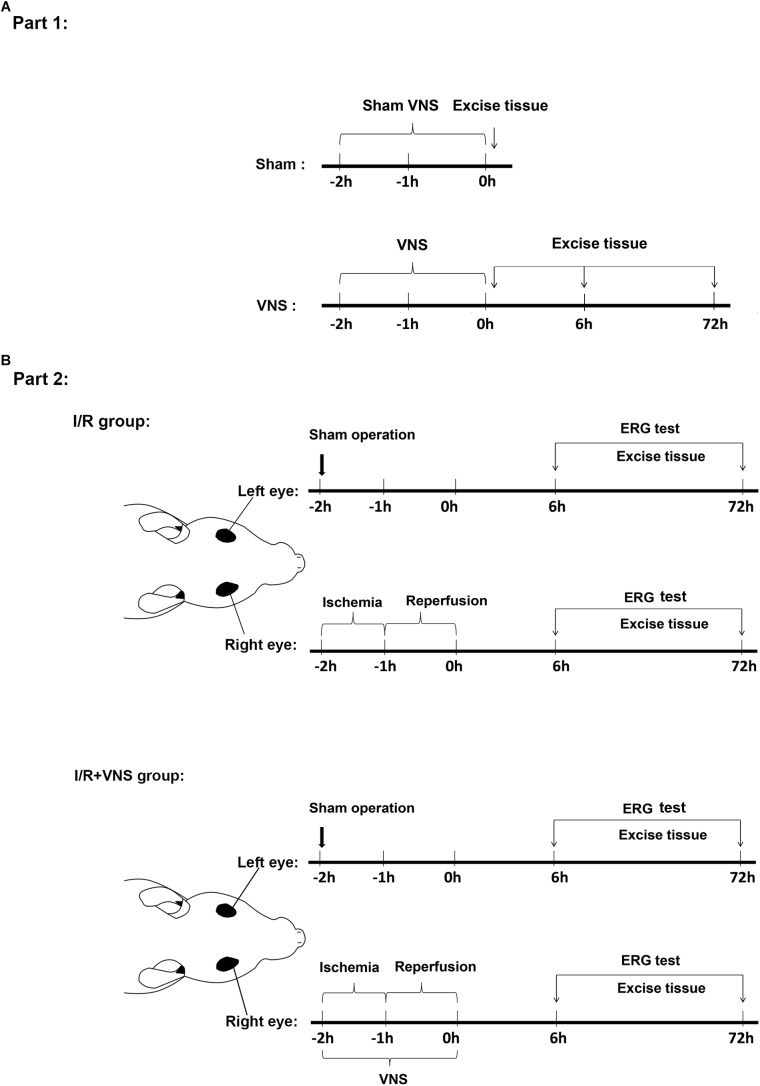
Timeline of the experimental design. **(A)** Experiments in the first part of the study detected whether VNS can activate the NOG-NTS-SSN-PPG neuron circuit. Rats were sacrificed at 0 h after sham VNS and at 0, 6, and 72 h after VNS. **(B)** Experiments in the second part of the study investigated the protective effect of VNS on retinal I/R injury. All operations were performed on the rat’s right eyes. The right vagal nerve was isolated and the stimulation was applied during the I/R period. Sham procedures were performed on the left eyes, which served as control. Rats were sacrificed at 6 and 72 h after reperfusion; (*n* = 4/group) I/R, ischemia/reperfusion; VNS, vagal nerve stimulation; ERG, electroretinogram.

Part 1: To detect the nodose ganglia (NOG), nucleus of the solitary tract (NTS), superior salivatory nucleus (SSN) and pterygopalatine ganglion (PPG) neural circuit, animals were randomly divided into 2 groups: (1) the sham group: sham right cervical VNS (*n* = 4) and (2) the VNS group: right cervical VNS for 2 h (*n* = 12, Figure 1A). At the end of the experiment, the brain, NOG (Calik et al., 2014) and PPG (Piagkou et al., 2012) tissues were removed, at 0 h after sham VNS and 0, 6, and 72 h after VNS, after transcranial perfusion with 100 ml of saline followed by 500 ml of 4% paraformaldehyde in 0.1 mol/L phosphate buffer at pH 7.4.

Part 2: To investigate the effect of VNS on retinal I/R injury, animals were randomly divided into two groups: (1) the I/R group with elevated IOP-induced ischemia for 1 h and then reperfusion for 1 h in the right eye (*n* = 16) and (2) the I/R + VNS group with right cervical VNS for 2 h during the retinal I/R period (*n* = 16). A sham procedure was performed on the left eyes of rats in both the I/R and I/R + VNS groups to serve as a control: a needle was inserted into the left anterior chamber without elevating the intraocular pressure. ERGs were performed before rats were sacrificed and the eyes and PPGs were harvested at 6 and 72 h after reperfusion (Figure 1B).

### Electroretinogram (ERG) Test

An ERG was performed at 6 and 72 h after retinal I/R injury. Rats were dark adapted for 4 h before recording and were anesthetized by xylazine and ketamine (i.p. injection, 10 and 100 mg/kg, respectively) and the pupils were dilated with 1% tropicamide. Then, 0.5% tetracaine hydrochloride was used to topically anesthetize the corneas for the duration under dim red light.

Electroretinograms were recorded using the RETIport 32 system (Roland Consult, Brandenburg, Germany) and gold-plated wire loop electrodes on the corneal surface as active electrodes. Stainless steel needle electrodes were inserted into the skin between the two ears and into the tail, serving as reference and ground leads, respectively. For scotopic ERG, responses to white flashes of 0.3, 1.0, and 3.0 cd⋅s/m^2^ were recorded, and the oscillatory potential (OP) was recorded at 3.0 cd⋅s/m^2^.

### Labeling Retinal Ganglion Cells

Rats were deeply anesthetized with xylazine and ketamine (i.p. injection, 10 and 100 mg/kg, respectively). Their eyes were then enucleated and fixed with 4% paraformaldehyde for 2 h. According to previous studies (Sánchez-Migallón et al., 2016; Wang S. et al., 2018), the whole mount retina was isolated and 4 radial incisions were made to create a petal shape to flatten the retina on glass. Retinas were later blocked in 5% bovine serum albumin (BSA) buffer and 0.2% Triton X-100 in PBS for 1 h at room temperature. The tissues were incubated overnight with anti-Brn3a antibody (Millipore Sigma, Billerica, MA, United States 1:200). Retinas were incubated with Cy3-conjugated secondary antibodies for 3 h at room temperature. These stained retinas were mounted in anti-fade mounting medium, and images were captured using a fluorescence microscope. The number of RGCs on each slide was counted by the Image-Pro plus 6.0 system (Media Cybernetics Inc., Bethesda, MD, United States).

### Western Blot Analysis

Retina samples were collected at 6 and 72 h after I/R. The proteins were extracted using RIPA buffer and separated by SDS gel. The membranes were incubated with primary antibody overnight at 4°C in TNF-α (Beyotime Institute of Biotechnology, Haimen, China, bs-2081R, 1:500), VIP (Beyotime Institute of Biotechnology, Haimen, China, bs-0077R, 1:500) and β-actin (Sigma-Aldrich, Taufkirchen, Germany, 1:5000). Specific protein levels were normalized to β-actin levels.

### Immunohistochemistry

According to a rat brain atlas (Watson, 2004), brains were divided into three blocks. Blocks containing the SSN and NTS were also embedded in paraffin. PPG samples were embedded in paraffin. Each of the paraffin blocks was sectioned in 5 μm-slices. The SSN and NTS were mounted on poly-lysine coated slides, deparaffinized and rehydrated sequentially. All tissues were washed with PBS, then were permeabilized in PBS containing 0.1% Triton X-100 and BSA for 1 h. Subsequently, tissues were incubated with primary antibody overnight at 4°C in c-fos (Servicebio, Wuhan, China, GB11069, 1:200) and VIP (Beyotime Institute of Biotechnology, Haimen, China, bs-0077R, 1:500). Tissue sections were mounted on glass slides and studied with a light microscope.

### Statistical Analysis

All data are presented as means ± SD. Statistical analysis was performed using *t*-tests for differences in ERG a- and b-wave ratios; one-way ANOVAs were used for the rest of the analyses using SPSS 16.0 software (SPSS Inc., Chicago, IL, United States). Bonferroni’s *post hoc* test was applied when a significant difference was found. *P* < 0.05 was considered to be statistically significant.

## Results

### Part 1: Effect of VNS on NOG-NTS-SSN-PPG Neural Pathway Activation

#### VNS Increases C-Fos Expression in NOG-NTS-SSN-PPG Neurons

**FIGURE 2:**
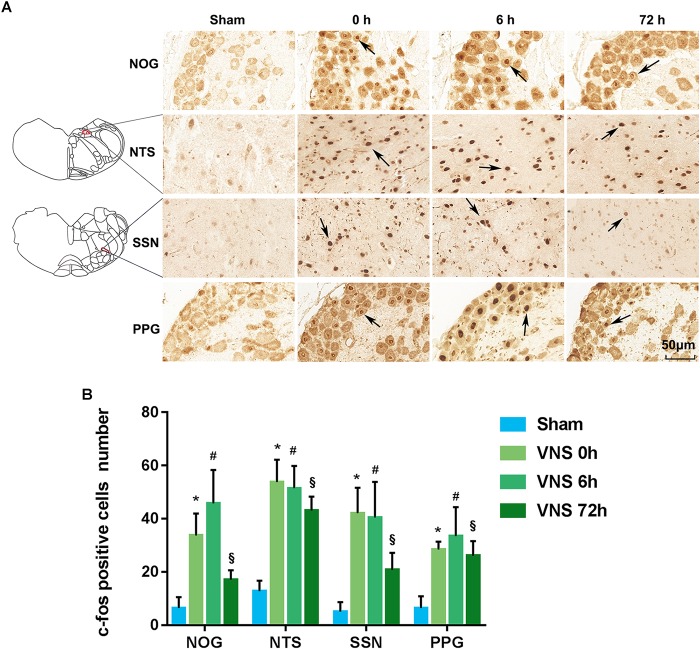
The expression of c-fos was evaluated. **(A)** NTS located at –13.32 mm from bregma, –4.32 mm from interaural and SSN located at –11.04 mm from bregma, –2.04 mm from interaural. C-fos positive cells in the NOG, NTS, SSN, and PPG are indicated by black arrows. The scale bar represents 50 μm. **(B)** Quantification of c-fos positive cell numbers in the NOG, NTS, SSN, and PPG tissues (*p* < 0.05); ^∗^*p* < 0.05 vs. VNS 0 h, ^#^*p* < 0.05 vs. VNS 6 h, ^§^ *p* < 0.05 vs. VNS 72 h; (*n* = 4/group); NOG, nodose ganglia; NTS, nucleus of solitary tract; SSN, superior salivatory nucleus; PPG, pterygopalatine ganglion.

C-fos is a classical marker used to indicate the activation of neurons. Thus, to investigate neural pathway activation, the expressions of c-fos in the NTS, SSN, NOG, and PPG sections were analyzed by immunohistochemistry (*n* = 4/group). Figure 2A shows the position of c-fos-positive cells in the NOG, NTS, SSN, and PPG of the sham group and VNS group. Figure 2B shows that the number of c-fos-positive cells of all tissues was significantly increased at 0, 6, and 72 h after VNS treatment compared to that in the sham group (*p* < 0.05).

### Part 2: Protective Effects of VNS on Retinal I/R Injury

#### VNS Alleviates Retinal Dysfunction

**FIGURE 3:**
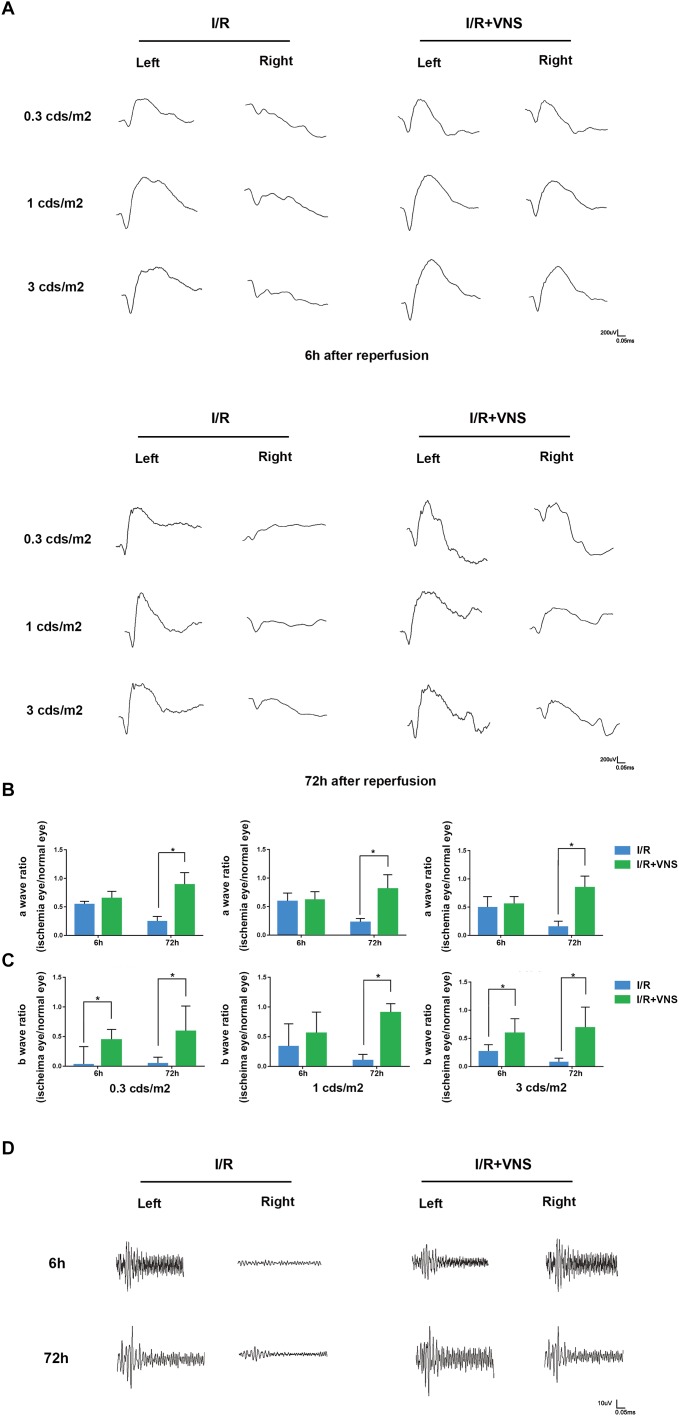
Flash electroretinograms were recorded at 6 and 72 h after I/R injury. **(A)** Representative traces from left and right eyes in the I/R group and I/R+VNS group at 6 and 72 h after reperfusion and at different luminance intensities. **(B,C)** Show the hypertensive/control amplitude ratios of a- and b-waves at 6 and 72 h at luminance intensities of 0.3, 1, and 3 cd⋅s/m^2^. **(D)** Representative oscillatory potential traces from the left and right eyes of the I/R and I/R+VNS groups at 6 and 72 h after reperfusion and at luminance intensity of 3 cd⋅s/m^2^; ^∗^*p* < 0.05; (*n* = 4/group).

To assess retinal function, scotopic ERG was recorded, and the a-wave and b-wave amplitude ratios (the amplitude of the ischemia (right) eye divided by the amplitude of the normal (left) eye) and the sum of the OP were analyzed at 6 and 72 h after reperfusion (*n* = 4/group). Compared with that in the left eyes, the a-wave amplitude significantly decreased after I/R injury (Figure 3A,B). After VNS treatment, the a-wave amplitude ratio significantly increased at luminance intensities of 0.3, 1, and 3 cd⋅s/m^2^, compared to the I/R group at 72 h following reperfusion (*p* < 0.05) (Figure 3B). Nevertheless, there was no significant difference in the a-wave amplitude ratio between the I/R group and the VNS group at 6 h following reperfusion at each luminance intensity.

Similarly, I/R significantly decreased the b-wave amplitude compared with that in the normal eyes. However, after VNS, there was a significant increase in the b-wave amplitude ratio compared to that in the I/R group at 72 h following reperfusion at each luminance intensity and at 6 h after reperfusion at 0.3 and 3 cd⋅s/m^2^ (*p* < 0.05; Figure 3C).

The sum of the OP amplitude was decreased in the I/R eyes compared to the normal eyes. However, in the I/R+VNS group, the sum of the OP amplitudes was increased at 6 and 72 h after reperfusion (Figure 3D).

#### Protective Effect of VNS on RCG Numbers in Retina

**FIGURE 4:**
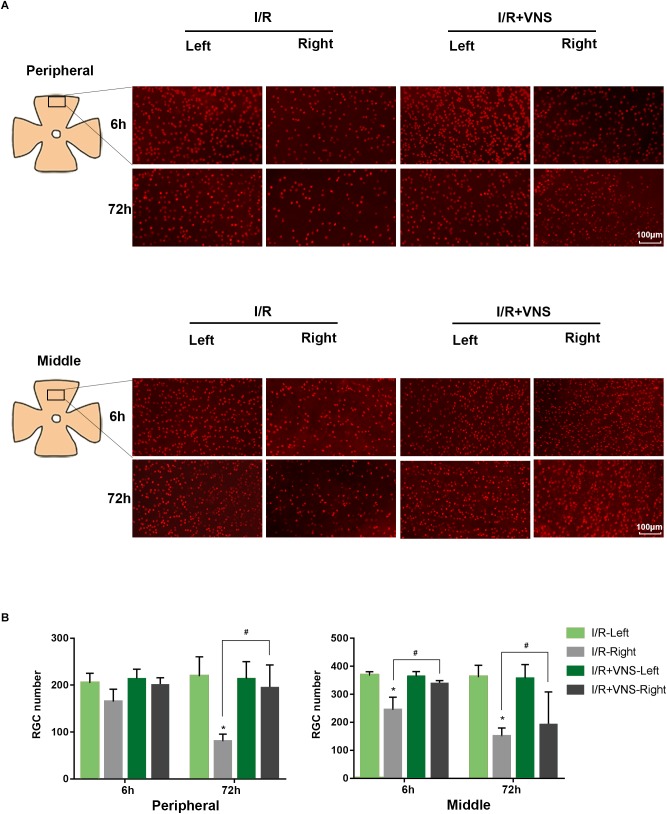
Retina ganglion cells were stained with Brn3a (red), and the number of Brn3a-positive cells was determined. **(A)** Representative images of the middle and peripheral areas of the retina showing Brn3a-positive cells in the left and right retinas of the I/R and I/R +VNS groups, at different time points. The scale bar represents 100 μm. **(B)** Quantification of RGC numbers in the middle and peripheral areas of the retina; ^∗^*p* < 0.05 vs. I/R-Left and I/R + VNS-Left groups; ^#^*p* < 0.05 vs. I/R +VNS-Right group; (*n* = 4/group).

To assess cell loss in the retina, RGCs were labeled with anti-Brn3a antibody and automatically counted (Figure 4). In the peripheral retina, I/R induced a reduction in RGC number at 72 h compared with that in normal eyes (*p* < 0.01), however, there were no significant changes in RGC number at 6 h. VNS significantly preserved the number of Brn3a-positive cells compared with that in the I/R retinas at 72 h after reperfusion (*p* < 0.01). Likewise, in the middle retina, there was a significant decrease in RGC number in the I/R retinas at both the 6 and 72 h time points, compared to that in the left retinas (*p* < 0.01). However, compared to that in the retinas in I/R group, the number of RGCs was preserved by VNS at 6 and 72 h (*p* < 0.01, Figure 4).

#### VNS Downregulates the Expression of TNF-α in the Retina

**FIGURE 5:**
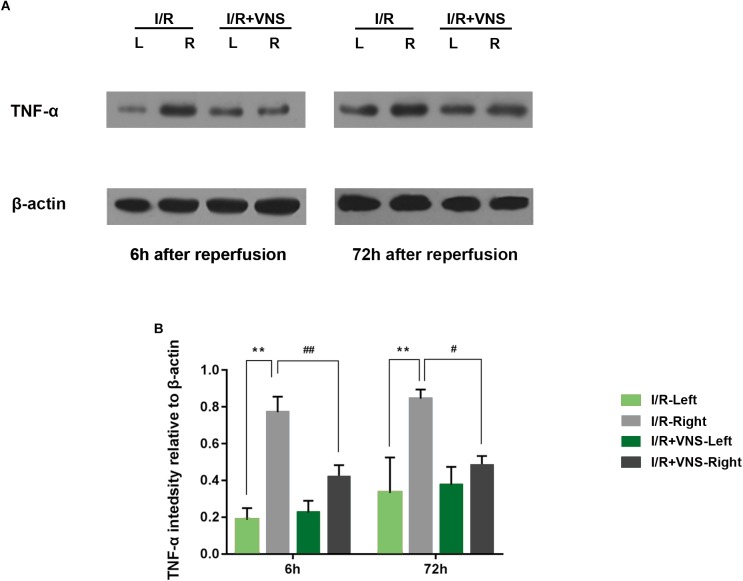
TNF-α expression in the retina was evaluated. **(A)** Representative TNF-α and β-actin bands. **(B)** Quantitative analysis of the protein expression level of TNF-α; ^∗∗^*p* < 0.01 vs. I/R-Left and I/R +VNS-Left groups; ^#^*p* < 0.05 and ^##^*p* < 0.01 vs. I/R +VNS-Right group; (*n* = 4/group).

The expression of TNF-α in the total retina was detected. Relative changes in protein levels in each group were calculated, normalizing to β-actin levels. Significantly higher levels of TNF-α were observed at 6 and 72 h following reperfusion than in the control retina. However, the expression of TNF-α was significantly decreased by VNS compared to that in retinas of the I/R group (Figure 5).

#### VNS Induces VIP Expression in the Retina and PPG

**FIGURE 6:**
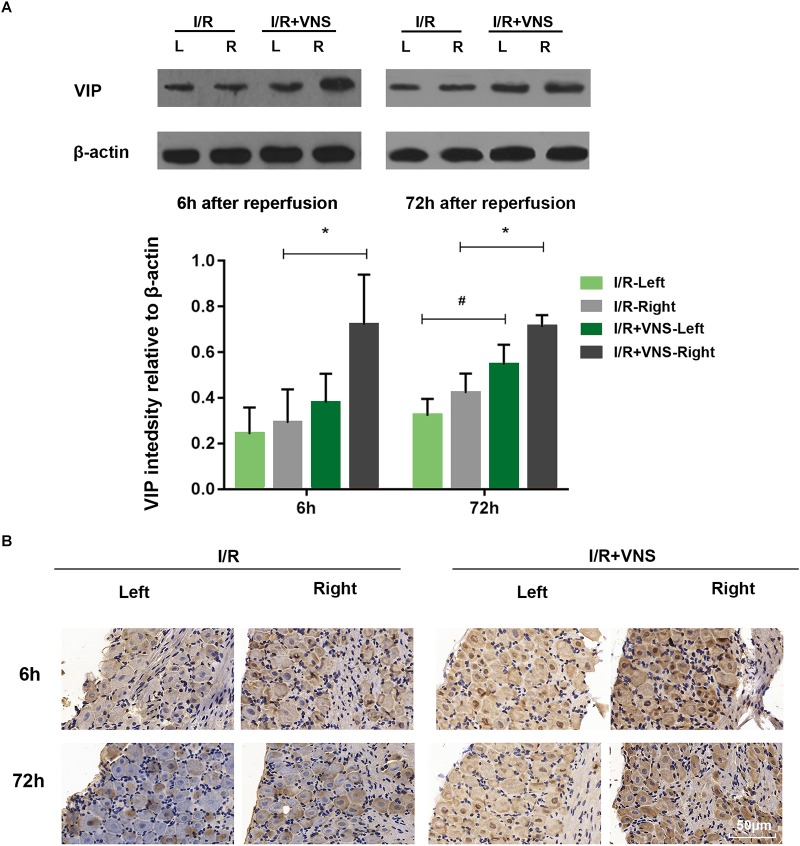
**(A)** Representative bands and quantitative analysis of VIP expression in the retina. **(B)** VIP expression in PPG as shown by immunohistochemistry. The scale bar represents 50 μm; ^∗^*p* < 0.05 vs. the I/R +VNS-Right group; ^#^*p* < 0.05 vs. the I/R +VNS-Left group; (*n* = 4/group).

The expression of VIP was evaluated by Western blot in the total retina (Figure 6A, *n* = 4/group). Compared with those in I/R retinas, VIP protein levels were significantly increased by VNS at 6 h (*p* = 0.005) and 72 h (*p* = 0.001) after reperfusion. When compared with I/R-left retinas, a significant difference was found in the I/R+VNS-left retina of VIP expression at 72 h (*p* = 0.044). However, there was no significant difference in VIP expression between I/R and control retina A similar trend was noticed for VIP expression as detected by the immunohistochemistry results (Figure 6B).

## Discussion

### Major Findings

**FIGURE 7:**
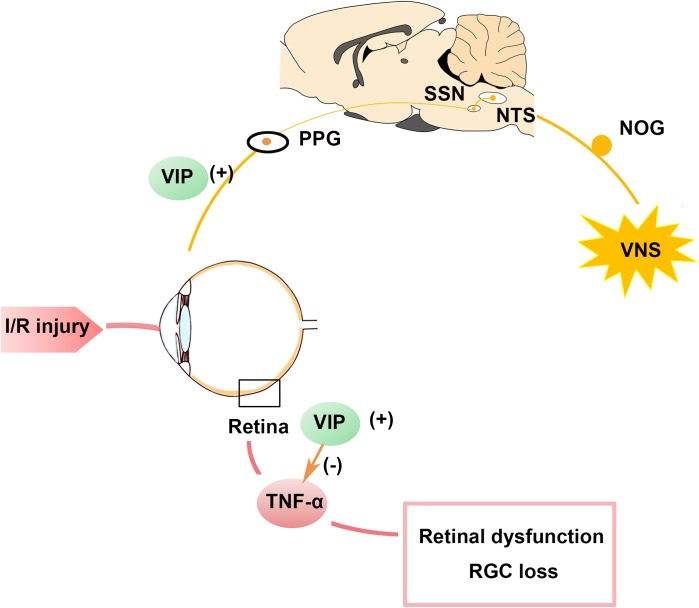
Schematic summary of the protective role of VNS in the ocular hypertension model. Stimulation of the vagal nerve can activate the NTS-SSN-PPG neural circuit and increase VIP expression in the PPG and retina. Increased VIP expression can attenuate the increase in TNF-α expression induced by retinal I/R injury and contributes to preserving the RGC number and improving retina function. I/R, ischemia/reperfusion; NOG, nodose ganglia; NTS, nucleus of solitary tract; PPG, pterygopalatine ganglion; RGC, retinal ganglion cell; SSN, superior salivatory nucleus; TNF-α, tumor necrosis factor-α; VIP, vasoactive intestinal polypeptide; VNS, vagal nerve stimulation.

In the present study, we present novel evidence that VNS can protect retinal function and preserve RGC numbers after I/R injury by downregulating the expression of pro-inflammatory factor. Moreover, we showed that activation of the NOG-NTS-SSN-PPG neural circuit and the release of neuropeptide VIP in the PPG and retina may mediate the protective effects of VNS during retinal I/R injury (Figure 7).

### Retinal Dysfunction and RGC Loss During I/R Injury

It has been well demonstrated that pro-inflammatory cytokines play a key role in retina I/R injury (Guo et al., 2016; Rivera and Dabouz, 2017). The level of TNF-α, a pro-inflammatory factor produced by leukocytes and microglia at the early stage of retinal I/R injury, was significantly elevated at 6, 12, and 24 h after reperfusion (Fontaine et al., 2002; Kim et al., 2017). Numerous studies have suggested that retinal morphology and functional changes are induced after cytokines are increased. ERG is a sensitive method for determining retinal function. The a-wave amplitude is related to photoreceptors in the olfactory nerve layer, while the b-wave originates from bipolar and Müller cells in the inner nuclear layer. The OP amplitude is triggered by amacrine cells (Weymouth and Vingrys, 2008). It has been reported that the a-wave, b-wave, and OP amplitude are significantly decreased, and the inflammatory response is simultaneously increased after I/R injury (Chen Y.J. et al., 2015; Fan, 2016; Yan, 2017). Similarly, our experiments support these reports as they have shown that the a-wave, b-wave, and OP amplitudes significantly decreased after retinal I/R injury, while the TNF-α level was increased.

### Possible Mechanisms Underlying the Protective Effects of VNS on the Retina

Vagal nerve stimulation is widely applied to attenuate I/R injury in multiple organs, including the myocardium (Zhao et al., 2013), kidney (Inoue et al., 2016), and cerebrum (Jiang et al., 2014) via anti-inflammatory responses. In accordance with these findings, the present study showed that VNS could preserve retinal morphology and function against I/R injury and downregulate TNF-α expression. Compared with those in the I/R group, a-wave, b-wave, and OP amplitudes were significantly increased by VNS which indicated the reversion of the photoreceptor, amacrine cell and bipolar cell function. In addition, the RGC number was preserved by VNS compared to that in the I/R group. These results further expanded the indications for the application of VNS.

It has been well demonstrated that NTS receives its preganglionic input from the NOG, which is the sensory afferent neuron body of the vagal nerve trunk and sends postganglionic fibers to the SSN. The SSN projects preganglionic fibers via the greater petrosal nerve to the PPG, which innervates the choroid and retina via parasympathetic nerves (Agassandian et al., 2002; Li et al., 2010; McDougal and Gamlin, 2015). Some studies have supported the existence of the NTS -SSN-PPG neural pathway. Spencer et al. (1990) observed pseudorabies virus-positive neurons in the NTS after direct injection of the virus in the PPG. Similar results have been reported by other researchers. Li et al. (2010) reported that positive cells were noted in the NTS after retrograde tracer biotinylated dextran amine 3K injection into the SSN; similarly positive cells were noted in the SSN following injection of the anterograde tracer biotinylated dextran amine 10K into the NTS. Agassandian et al. (2002) also found positive cells in the NTS after retrograde tracer wheat germ agglutinin-horseradish peroxidase injection into the SSN. The results from the first part of this study showed that the expression of c-fos, a marker used to indicate the activation of neurons, was significantly increased in the NTS, SSN and PPG tissues at 0, 6, and 72 h after VNS, compared to the sham group. Together with the anatomical evidence, our results validate the idea that VNS could activate the NOG-NTS-SSN-PPG neural circuit and contribute to autonomic regulation in peripheral tissues.

Studies have revealed that nearly 40% of the neurons in the PPG are VIP-positive neurons (Szczurkowski et al., 2013). In addition, Li C et al. (Li et al., 2010, 2015, 2016a,b) reported that NTS stimulation can activate the SSN-PPG neural pathway and increase VIP expression in the choroid. VIP is a well-known neuroprotective peptide and its primary function is in the anti-inflammatory response. It has been reported that VIP can downregulate pro-inflammatory cytokines and mediators, and can promote the expression of neurotrophic factors (such as brain-derived neurotrophic factor) to prevent inflammation-induced neuronal loss (Delgado, 2003; Fernandez-Martin et al., 2006). Tuncel et al. (1996) and Szabadfi et al. (2012) reported that VIP administration can protect the retina against I/R injury and preserve retinal structure. In addition, Shi et al. reported that VIP could protect human retinal microvascular endothelial cells against high glucose-induced endothelial dysfunction via reducing TNF-α expression (Shi et al., 2016). More importantly, it has been demonstrated that VNS could promote the release of endogenous VIP (Havel et al., 1997; Henning and Sawmiller, 2001). In accordance with these studies, we found that VIP expression in the PPG and retina significantly increased after VNS. Meanwhile, the expression of TNF-α was significantly decreased after VNS. Together with the results from the first part of the study, those from the second part of the study demonstrated that VNS could protect against retinal I/R injury by activating the NOG-NTS-SSN-PPG neural circuit and promoting the release of the anti-inflammatory neuropeptide VIP in the PPG and retina. The released VIP can then contribute to attenuation of the inflammatory response and, thus, to retinal I/R injury. Several studies (Zhang and Osborne, 2006; Wang W. et al., 2018) reported that unilateral stimulation-induced ipsilateral activation or the release of certain substances are have more than a contralateral effect. Our results show the consisted findings that the expression of VIP was increased in the left retina and PPG of I/R+VNS group but less than in the right retina.

### Clinical Implication

Retinal I/R injury is a well-known pathological process associated with retinal disease, such as glaucoma and diabetic retinopathy, which can cause RGCs and vision loss (Goldblum and Mittag, 2002; Osborne et al., 2004). These neuronal damages occur at the early stage of these diseases, which reveals the newer perspective of neuroprotective therapy (Nafissi and Foldvari, 2015; Nuzzi and Tridico, 2017). However, there is currently no effective treatment for neuronal damage. The present study suggests that VNS could protect against retinal I/R injury. Recently, transcutaneous electrical stimulation of the auricular branch of the vagal nerve has been demonstrated to be as safe and effective as cervical VNS (He et al., 2013; Yu et al., 2013; Stavrakis et al., 2015). Therefore, based on our present experiment and further research, transcutaneous electrical stimulation of the auricular branch of the vagal nerve may become a novel therapeutic approach for attenuating retinal I/R injury.

### Study Limitations

The present study has several limitations. First, previous studies have reported that the frequency of cervical VNS used in different I/R injuries ranges from 2 to 20 Hz (Cao et al., 2017; Stauss, 2017; Mirza et al., 2018). In the present study, we evaluated the effect of VNS at 5 Hz in the retinal I/R model. Additional studies are required to explore the best parameters of VNS for the prevention of retinal I/R injury. Secondly, we only followed the animals for 72 h after reperfusion, however, a longer follow-up period could be informative. Thirdly, the classical cholinergic anti-inflammatory effect of VNS was mainly mediated by the α7 nicotinic acetylcholine receptor. Further experiments are needed to clarify the role of this receptor relation to the protective effects of VNS on retinal I/R injury. Fourthly, for the purpose of decreasing the influence of individual differences and the consideration of animal welfare, contralateral un-injured eyes of I/R rats as a control group was used in the present study as Chi et al. (2014) previously described.

## Data Availability Statement

All datasets (generated/analyzed) for this study are included in the manuscript.

## Author Contributions

M-NJ, Y-YZ, M-LH, and Y-QX designed this experiment, critically revised it, and finally approved the version to be published. D-HH and J-YY took part in the operation, for example, western blotting, ERG test, and the rat model establishment. M-NJ drafted and revised the manuscript and took part in a majority of the work. All authors read and approved the manuscript to be published.

## Conflict of Interest Statement

The authors declare that the research was conducted in the absence of any commercial or financial relationships that could be construed as a potential conflict of interest.

## Abbreviations

I/R: ischemia/reperfusion
IOP: intraocular pressure
NOG: nodose ganglia
NTS: nucleus of solitary tract
PPG: pterygopalatine ganglion
RGC: retinal ganglion cell
SSN: superior salivatory nucleus
TNF-α: tumor necrosis factor-α
VIP: vasoactive intestinal polypeptide
VNS: vagal nerve stimulation

## References

[B1] Adams C. M. Stacy R. Rangaswamy N. Bigelow C. Grosskreutz C. L. Prasanna G. (2018). Glaucoma - next generation therapeutics: impossible to possible. *Pharm. Res.* 36:25. 10.1007/s11095-018-2557-4 30547244

[B2] Agassandian K. Fazan V. P. Adanina V. Talman W. T. (2002). Direct projections from the cardiovascular nucleus tractus solitarii to pontine preganglionic parasympathetic neurons: a link to cerebrovascular regulation. *J. Comp. Neurol.* 452 242–254. 10.1002/cne.10372 12353220

[B3] Agassandian K. Fazan V. P. S. Margaryan N. Dragon D. N. Riley J. Talman W. T. (2003). A novel central pathway links arterial baroreceptors and pontine parasympathetic neurons in cerebrovascular control. *Cell. Mol. Neurobiol.* 23 463–478. 10.1023/A:1025059710382 14514008 PMC11530209

[B4] Atlasz T. Werling D. Song S. Szabo E. Vaczy A. Kovari P. (2018). Retinoprotective effects of TAT-bound vasoactive intestinal peptide and pituitary adenylate cyclase activating polypeptide. *J. Mol. Neurosci.* 10.1007/s12031-018-1229-5 [Epub ahead of print]. 30542799 PMC6581923

[B5] Cakmak A. I. Basmak H. Gursoy H. Ozkurt M. Yildirim N. Erkasap N. (2017). Vasoactive intestinal peptide, a promising agent for myopia? *Int. J. Ophthalmol.* 10 211–216. 10.18240/ijo.2017.02.05 28251078 PMC5313542

[B6] Calik M. W. Radulovacki M. Carley D. W. (2014). A method of nodose ganglia injection in sprague-dawley rat. *J. Vis. Exp.* 93:e52233. 10.3791/52233 25490160 PMC4354328

[B7] Cao J. Lu K. H. Powley T. L. Liu Z. (2017). Vagal nerve stimulation triggers widespread responses and alters large-scale functional connectivity in the rat brain. *PLoS One* 12:e0189518. 10.1371/journal.pone.0189518 29240833 PMC5730194

[B8] Chandrasekharan B. Nezami B. G. Srinivasan S. (2013). Emerging neuropeptide targets in inflammation: NPY and VIP. *Am. J. Physiol. Gastrointest. Liver Physiol.* 304 949–957. 10.1152/ajpgi.00493.2012 23538492 PMC3680683

[B9] Chen L. Yuan W. Chen Z. Wu S. Ge J. Chen J. (2015). Vasoactive intestinal peptide represses activation of tumor-associated macrophages in gastric cancer via regulation of TNFalpha, IL-6, IL-12 and iNOS. *Int. J. Oncol.* 47 1361–1370. 10.3892/ijo.2015.3126 26314485

[B10] Chen Y. J. Huang Y. S. Chen J. T. Chen Y. H. Tai M. C. Chen C. L. (2015). Protective effects of glucosamine on oxidative-stress and ischemia/reperfusion-induced retinal injury. *Invest. Ophthalmol. Vis. Sci.* 56 1506–1516. 10.1167/iovs.14-15726 25655796

[B11] Chi W. Li F. Chen H. Wang Y. Zhu Y. Yang X. (2014). Caspase-8 promotes NLRP1/NLRP3 inflammasome activation and IL-1beta production in acute glaucoma. *Proc. Natl. Acad. Sci. U.S.A.* 111 11181–11186. 10.1073/pnas.1402819111 25024200 PMC4121847

[B12] Chunyan L. Fitzgerald M. E. C. Nobel D. M. Sherry C. C. Ledoux M. S. Suzhen G. (2015). The identification and neurochemical characterization of central neurons that target parasympathetic preganglionic neurons involved in the regulation of choroidal blood flow in the rat eye using pseudorabies virus, immunolabeling and conventional pathway tracing methods. *Front. Neuroanat.* 9:65. 10.3389/fnana.2015.00065 26082687 PMC4451581

[B13] Delgado M. (2003). Vasoactive intestinal peptide and pituitary adenylate cyclase-activating polypeptide inhibit the production of inflammatory mediators by activated microglia. *J. Leukoc. Biol.* 73 155–164. 10.1189/jlb.0702372 12525573

[B14] Fan J. (2016). Suppression of acid sphingomyelinase protects the retina from ischemic injury. *Invest. Ophthalmol. Vis. Sci.* 57 4476–4484. 10.1167/iovs.16-19717 27571014 PMC5015980

[B15] Fang I. M. Yang C. M. Yang C. H. Chiou S. H. Chen M. S. (2013). Transplantation of induced pluripotent stem cells without C-Myc attenuates retinal ischemia and reperfusion injury in rats. *Exp. Eye Res.* 113 49–59. 10.1016/j.exer.2013.05.007 23726881

[B16] Fernandez-Martin A. Gonzalez-Rey E. Chorny A. Martin J. Pozo D. Ganea D. (2006). VIP prevents experimental multiple sclerosis by downregulating both inflammatory and autoimmune components of the disease. *Ann. N. Y. Acad. Sci.* 1070 276–281. 10.1196/annals.1317.026 16888178

[B17] Fontaine V. Mohand-Said S. Hanoteau N. Fuchs C. Pfizenmaier K. Eisel U. (2002). Neurodegenerative and neuroprotective effects of tumor Necrosis factor (TNF) in retinal ischemia: opposite roles of TNF receptor 1 and TNF receptor 2. *J. Neurosci.* 22:Rc216. 11917000 10.1523/JNEUROSCI.22-07-j0001.2002 PMC6758303

[B18] Ganea D. Hooper K. M. Kong W. (2015). The neuropeptide vasoactive intestinal peptide: direct effects on immune cells and involvement in inflammatory and autoimmune diseases. *Acta Physiol.* 213 442–452. 10.1111/apha.12427 25422088 PMC4484298

[B19] Goldblum D. Mittag T. (2002). Prospects for relevant glaucoma models with retinal ganglion cell damage in the rodent eye. *Vision Res.* 42 471–478. 10.1016/S0042-6989(01)00194-8 11853763

[B20] Guo Z. Yu S. Chen X. Ye R. Zhu W. Liu X. (2016). NLRP3 is involved in ischemia/reperfusion injury. *CNS Neurol. Disord. Drug Targets* 15 699–712. 10.2174/1871527315666160321111829 26996163

[B21] Hashem H. E. Abd El-Haleem M. R. Amer M. G. Bor’i A. (2017). Pomegranate protective effect on experimental ischemia/reperfusion retinal injury in rats (histological and biochemical study). *Ultrastruct. Pathol.* 41 346–357. 10.1080/01913123.2017.1346737 28796566

[B22] Havel P. J. Dunning B. E. Verchere C. B. Baskin D. G. O’Dorisio T. Taborsky G. J. Jr. (1997). Evidence that vasoactive intestinal polypeptide is a parasympathetic neurotransmitter in the endocrine pancreas in dogs. *Regul. Pept.* 71 163–170. 10.1016/S0167-0115(97)01014-8 9350974

[B23] He W. Jing X. H. Zhu B. Zhu X. L. Li L. Bai W. Z. (2013). The auriculo-vagal afferent pathway and its role in seizure suppression in rats. *BMC Neurosci.* 14:85. 10.1186/1471-2202-14-85 23927528 PMC3751281

[B24] Henning R. J. Sawmiller D. R. (2001). Vasoactive intestinal peptide: cardiovascular effects. *Cardiovasc. Res.* 49 27–37. 10.1016/S0008-6363(00)00229-7 11121793

[B25] Inoue T. Abe C. Sung S. S. Moscalu S. Jankowski J. Huang L. (2016). Vagus nerve stimulation mediates protection from kidney ischemia-reperfusion injury through alpha7nAChR+ splenocytes. *J. Clin. Invest.* 126 1939–1952. 10.1172/jci83658 27088805 PMC4855936

[B26] Jiang Y. Li L. Liu B. Zhang Y. Chen Q. Li C. (2014). Vagus nerve stimulation attenuates cerebral ischemia and reperfusion injury via endogenous cholinergic pathway in rat. *PLoS One* 9:e102342. 10.1371/journal.pone.0102342 25036185 PMC4103831

[B27] Kaur C. Foulds W. S. Ling E. A. (2008). Hypoxia-ischemia and retinal ganglion cell damage. *Clin. Ophthalmol.* 2 879–889. 10.2147/OPTH.S3361 19668442 PMC2699791

[B28] Kim C. R. Kim J. H. Park H. L. Park C. K. (2017). Ischemia reperfusion injury triggers TNFalpha induced-Necroptosis in rat retina. *Curr. Eye Res.* 42 771–779. 10.1080/02713683.2016.1227449 27732109

[B29] Le J. T. Rouse B. Gazzard G. (2018). Iridotomy to slow progression of visual field loss in angle-closure glaucoma. *Cochrane Database Syst. Rev.* 6:CD012270. 10.1002/14651858.CD012270.pub2 29897635 PMC6026549

[B30] Li C. Fitzgerald M. E. Del Mar N. Cuthbertson-Coates S. LeDoux M. S. Gong S. (2015). The identification and neurochemical characterization of central neurons that target parasympathetic preganglionic neurons involved in the regulation of choroidal blood flow in the rat eye using pseudorabies virus, immunolabeling and conventional pathway tracing methods. *Front. Neuroanat.* 9:65. 10.3389/fnana.2015.00065 26082687 PMC4451581

[B31] Li C. Fitzgerald M. E. Del Mar N. Reiner A. (2016a). Disinhibition of neurons of the nucleus of solitary tract that project to the superior salivatory nucleus causes choroidal vasodilation: implications for mechanisms underlying choroidal baroregulation. *Neurosci. Lett.* 633 106–111. 10.1016/j.neulet.2016.09.029 27663135 PMC5117681

[B32] Li C. Fitzgerald M. E. Del Mar N. Reiner A. (2016b). Stimulation of baroresponsive parts of the nucleus of the solitary tract produces nitric oxide-mediated choroidal vasodilation in rat eye. *Front. Neuroanat.* 10:94. 10.3389/fnana.2016.00094 27774055 PMC5053990

[B33] Li C. Fitzgerald M. E. Ledoux M. S. Gong S. Ryan P. Del Mar N. (2010). Projections from the hypothalamic paraventricular nucleus and the nucleus of the solitary tract to prechoroidal neurons in the superior salivatory nucleus: pathways controlling rodent choroidal blood flow. *Brain Res.* 1358 123–139. 10.1016/j.brainres.2010.08.065 20801105 PMC2949519

[B34] Li P. Liu H. Sun P. Wang X. Wang C. Wang L. (2016). Chronic vagus nerve stimulation attenuates vascular endothelial impairments and reduces the inflammatory profile via inhibition of the NF-kappaB signaling pathway in ovariectomized rats. *Exp. Gerontol.* 74 43–55. 10.1016/j.exger.2015.12.005 26692419

[B35] Liu L. Jiang Y. Steinle J. J. (2016). Compound 49b restores retinal thickness and reduces degenerate capillaries in the rat retina following ischemia/reperfusion. *PLoS One* 11:e0159532. 10.1371/journal.pone.0159532 27439004 PMC4954700

[B36] McDougal D. H. Gamlin P. D. (2015). Autonomic control of the eye. *Compr. Physiol.* 5 439–473. 10.1002/cphy.c140014 25589275 PMC4919817

[B37] Mi X. S. Feng Q. Lo A. C. Chang R. C. Lin B. Chung S. K. (2012). Protection of retinal ganglion cells and retinal vasculature by *Lycium barbarum* polysaccharides in a mouse model of acute ocular hypertension. *PLoS One* 7:e45469. 10.1371/journal.pone.0045469 23094016 PMC3477168

[B38] Mirza K. B. Alenda A. Eftekhar A. Grossman N. Nikolic K. Bloom S. R. (2018). Influence of cholecystokinin-8 on compound nerve action potentials from ventral gastric vagus in rats. *Int. J. Neural Syst.* 28:1850006. 10.1142/s0129065718500065 29631504

[B39] Munemasa Y. Kitaoka Y. (2012). Molecular mechanisms of retinal ganglion cell degeneration in glaucoma and future prospects for cell body and axonal protection. *Front. Cell. Neurosci.* 6:60. 10.3389/fncel.2012.00060 23316132 PMC3540394

[B40] Nafissi N. Foldvari M. (2015). Neuroprotective therapies in glaucoma: II. Genetic nanotechnology tools. *Front. Neurosci.* 9:355. 10.3389/fnins.2015.00355 26528114 PMC4604245

[B41] Narayanan S. P. Rojas M. Suwanpradid J. Toque H. A. Caldwell R. W. Caldwell R. B. (2013). Arginase in retinopathy. *Prog. Retin. Eye Res.* 36 260–280. 10.1016/j.preteyeres.2013.06.002 23830845 PMC3759622

[B42] Nuzzi R. Tridico F. (2017). Glaucoma: biological trabecular and neuroretinal pathology with perspectives of therapy innovation and preventive diagnosis. *Front. Neurosci.* 11:494. 10.3389/fnins.2017.00494 28928631 PMC5591842

[B43] Osborne N. N. Casson R. J. Wood J. P. Chidlow G. Graham M. Melena J. (2004). Retinal ischemia: mechanisms of damage and potential therapeutic strategies. *Prog. Retin. Eye Res.* 23 91–147. 10.1016/j.preteyeres.2003.12.001 14766318

[B44] Palmhof M. Lohmann S. Schulte D. Stute G. Wagner N. Dick H. B. (2018). Fewer functional deficits and reduced cell death after ranibizumab treatment in a retinal ischemia model. *Int. J. Mol. Sci.* 19:E1636. 10.3390/ijms19061636 29857531 PMC6032266

[B45] Piagkou M. Demesticha T. Troupis T. Vlasis K. Skandalakis P. Makri A. (2012). The pterygopalatine ganglion and its role in various pain syndromes: from anatomy to clinical practice. *Pain Pract.* 12 399–412. 10.1111/j.1533-2500.2011.00507.x 21956040

[B46] Pinar-Sueiro S. Hurtado J. ÁZ. Veiga-Crespo P. Sharma S. C. Vecino E. (2013). Neuroprotective effects of topical CB1 agonist WIN 55212-2 on retinal ganglion cells after acute rise in intraocular pressure induced ischemia in rat. *Exp. Eye Res.* 110 55–58. 10.1016/j.exer.2013.02.009 23454099

[B47] Rivera J. C. Dabouz R. (2017). Ischemic retinopathies: oxidative stress and inflammation. *Oxid. Med. Cell. Longev.* 2017:3940241. 10.1155/2017/3940241 29410732 PMC5749295

[B48] Sánchez-Migallón M. C. Valiente-Soriano F. J. Nadal-Nicolás F. M. Vidal-Sanz M. Agudo-Barriuso M. (2016). Apoptotic retinal ganglion cell death after optic nerve transection or crush in mice: delayed RGC loss with BDNF or a caspase 3 inhibitor. *Invest. Ophthalmol. Vis. Sci.* 57 81–93. 10.1167/iovs.15-17841 26780312

[B49] Shi H. Carion T. W. Jiang Y. Steinle J. J. Berger E. A. (2016). VIP protects human retinal microvascular endothelial cells against high glucose-induced increases in TNF-α and enhances RvD1. *Prostaglandins Other Lipid Mediat.* 123 28–32. 10.1016/j.prostaglandins.2016.03.001 27026343 PMC5242223

[B50] Spencer S. E. Sawyer W. B. Wada H. Platt K. B. Loewy A. D. (1990). CNS projections to the pterygopalatine parasympathetic preganglionic neurons in the rat: a retrograde transneuronal viral cell body labeling study. *Brain Res.* 534 149–169. 10.1016/0006-8993(90)90125-U 1705849

[B51] Stauss H. M. (2017). Differential hemodynamic and respiratory responses to right and left cervical vagal nerve stimulation in rats. *Physiol. Rep.* 5:e13244. 10.14814/phy2.13244 28400500 PMC5392529

[B52] Stavrakis S. Humphrey M. B. Scherlag B. J. Hu Y. Jackman W. M. Nakagawa H. (2015). Low-level transcutaneous electrical vagus nerve stimulation suppresses atrial fibrillation. *J. Am. Coll. Cardiol.* 65 867–875. 10.1016/j.jacc.2014.12.026 25744003 PMC4352201

[B53] Szabadfi K. Danyadi B. Kiss P. Tamas A. Fabian E. Gabriel R. (2012). Protective effects of vasoactive intestinal peptide (VIP) in ischemic retinal degeneration. *J. Mol. Neurosci.* 48 501–507. 10.1007/s12031-012-9774-9 22544514

[B54] Szczurkowski A. Sienkiewicz W. Kuchinka J. Kaleczyc J. (2013). Morphology and immunohistochemical characteristics of the pterygopalatine ganglion in the chinchilla (Chinchilla laniger, Molina). *Pol. J. Vet. Sci.* 16 359–368. 10.2478/pjvs-2013-0048 23971205

[B55] Tuncel N. Basmak H. Uzuner K. Tuncel M. Altiokka G. Zaimoglu V. (1996). Protection of rat retina from ischemia-reperfusion injury by vasoactive intestinal peptide (VIP): the effect of VIP on lipid peroxidation and antioxidant enzyme activity of retina and choroid. *Ann. N. Y. Acad. Sci.* 805 489–498. 10.1111/j.1749-6632.1996.tb17509.x 8993429

[B56] Wang S. Chen L. Zhang P. Wang X. Sun Y. Ma L. (2018). Transplantation of retinal progenitor cells from optic cup-like structures differentiated from human embryonic stem cells in vitro and in vivo generation of retinal ganglion-like cells. *Stem Cells Dev.* 10.1089/scd.2018.0076 [Epub ahead of print]. 30526386

[B57] Wang W. Zhong X. Li Y. Guo R. Du S. Wen L. (2018). Rostral ventromedial medulla-mediated descending facilitation following P2X7 receptor activation is involved in the development of chronic postoperative pain. *J. Neurochem.* 10.1111/jnc.14650 [Epub ahead of print]. 30570747

[B58] Watson C. (2004). *The Rat Brain in Stereotaxic Coordinates-The New Coronal Set.* Cambridge, MA: Academic press.

[B59] Weymouth A. E. Vingrys A. J. (2008). Rodent electroretinography: methods for extraction and interpretation of rod and cone responses. *Prog. Retin. Eye Res.* 27 1–44. 10.1016/j.preteyeres.2007.9.003 18042420

[B60] Xu J. Kong X. Xiu H. Dou Y. Wu Z. Sun P. (2018). Combination of curcumin and vagus nerve stimulation attenuates cerebral ischemia/reperfusion injury-induced behavioral deficits. *Biomed. Pharmacother.* 103 614–620. 10.1016/j.biopha.2018.04.069 29677548

[B61] Yan H. (2017). The protective effects of αB-Crystallin on ischemia-reperfusion injury in the rat retina. *J. Ophthalmol.* 2017:7205408. 10.1155/2017/7205408 29098085 PMC5643040

[B62] Yu L. Scherlag B. J. Li S. Fan Y. Dyer J. Male S. (2013). Low-level transcutaneous electrical stimulation of the auricular branch of the vagus nerve: a noninvasive approach to treat the initial phase of atrial fibrillation. *Heart Rhythm.* 10 428–435. 10.1016/j.hrthm.2012.11.019 23183191

[B63] Zhao M. He X. Bi X. Y. Yu X. J. Gil Wier W. Zang W. J. (2013). Vagal stimulation triggers peripheral vascular protection through the cholinergic anti-inflammatory pathway in a rat model of myocardial ischemia/reperfusion. *Basic Res. Cardiol.* 108:345. 10.1007/s00395-013-0345-1 23519622

[B64] Zhang B. Osborne N. N. (2006). Oxidative-induced retinal degeneration is attenuated by epigallocatechin gallate. *Brain Res.* 1124 176–187. 10.1016/j.brainres.2006.09.067 17084820

[B65] Zheng L. Gong B. Hatala D. A. Kern T. S. (2007). Retinal ischemia and reperfusion causes capillary degeneration: similarities to diabetes. *Invest. Ophthalmol. Vis. Sci.* 48 361–367. 10.1167/iovs.06-0510 17197555

